# Exploring the Impact of Blood Disorders on Dental Caries

**DOI:** 10.7759/cureus.47159

**Published:** 2023-10-16

**Authors:** Gayatri Kale, Vidya Maheswari Nelakurthi, Priyanka Paul

**Affiliations:** 1 Public Health Dentistry, Sharad Pawar Dental College and Hospital, Datta Meghe Institute of Higher Education and Research, Wardha, IND

**Keywords:** iron deficiency anemia, dental caries, sickle cell anemia, leukemia, thalassemia

## Abstract

Blood comprises various cellular elements and serves as our immune system's second line of defense. Deviations from its normal composition can have adverse effects on health. At the same time, the oral mucosa in the oral cavity functions as the body's first line of defense, and any anomalies or diseases within it can give rise to both systemic and oral complications. If left untreated, caries can lead to severe tooth damage or extraction, potentially affecting an individual's nutrition and overall health. This review article focuses on the importance of understanding the intricate relationship between blood disorders and oral health. It underscores the profound impact of oral manifestations of blood disorders such as β-thalassemia, sickle cell disease, iron deficiency anemia, leukemia, hemophilia, Plummer-Vinson syndrome, erythroblastosis fetalis, Fanconi anemia, cyclic neutropenia, and acute lymphoblastic leukemia on the overall well-being of an individual.

## Introduction and background

The World Health Organization emphasizes that an individual's health and overall well-being is significantly influenced by oral health. Examining the oral cavity offers valuable insights into one's general health, as its condition mirrors and affects our overall bodily well-being. As the oral cavity acts as the body's main line of defense through the oral mucosa, many oral disorders and severe systemic diseases have similar risk factors. Dental decay, periodontal disease, gingivitis, and oral cancer are some of the most prevalent conditions that pose significant threats to oral health [1-3]. The oral cavity plays host to a rich and varied array of bacteria within the body, including those that can exacerbate general health issues when coupled with dental decay and periodontal disease [4].

In an epidemiological study conducted in 2019 among children aged 6-12 years in orphanages in Kerala, India, the prevalence of dental caries was 77.44% [5]. A hospital-based case-control study was conducted in Pune, India, involving individuals who smoked more than 10 *bidis*/cigarettes daily for over 25 years. The prevalence of oral cancer in this group was 95%, indicating an elevated risk [6]. A systematic review and meta-analysis conducted by Janakiram et al. in 2020 involved an electronic search, which revealed a prevalence of 51% for periodontal disease and 46.6% for gingivitis. Mild to moderate periodontitis was seen in 26.2%, while severe periodontitis was seen in 19% of cases [7].

Periapical infections and pulpitis can result from dental caries when infection spreads from carious enamel and dentin into the pulp. Pulp removal becomes essential to stop bacteria from spreading further. If left untreated, an infected, decaying tooth may progress to the point where extraction is necessary. Oral diseases, including periodontal disease, infected root canals, cavitations in extraction sites, and other conditions that impact the oral cavity, such as oral cancer, notably influence general health. A study conducted by Watanabe et al. in 2020 showed that deterioration in masticatory function is associated with deterioration in general health, which has been attributed to deterioration in the nutritional status [8].

Blood is a unique type of tissue consisting of plasma and various types of blood cells, each with its specific role and structure. These blood cells are produced in the bone marrow. Among these cells, erythrocytes, which have a distinctive biconcave shape and are responsible for transporting oxygen, typically range from four to six million cells per microliter. Another crucial component is leukocytes, commonly known as white blood cells (WBCs), with a typical count of about 4000-11,000 cells/µL. WBCs serve as the body's defense against diseases by protecting it from harmful pathogens and toxic substances. Usually, there are more red blood cells than white blood cells in the blood, but any deviation from these standard counts can affect an individual's overall health.

Various factors, including salivary flow, biofilm formation, diet, substrate, and oral hygiene practices, influence the occurrence of dental caries. Saliva plays a pivotal role in maintaining oral health by exerting a cleansing effect on tooth surfaces, possessing anti-cariogenic properties, offering buffering capacity, exhibiting proteolytic activity and antibacterial capabilities, and regulating the processes of demineralization and remineralization. These combined mechanisms work in concert to help prevent the development of caries. A reduction in salivary secretion can lead to a decrease in anti-cariogenic and proteolytic activity [9]. Dental plaque is clinically characterized as a firm, yellow-greyish material that adheres stubbornly to intraoral hard surfaces, encompassing healthy tooth structures and removable and fixed dental restorations. Elevated plaque levels contribute to the accumulation of bacteria and the onset of periodontal diseases, increasing the likelihood of developing cavities [10]. Within this plaque, various bacteria can accumulate, including *Streptococcus mutans*, *Staphylococcus aureus*, *Actinomyces*, *Neisseria*, and other species [11]. This bacterial buildup can lead to a drop in pH levels below 5.5, demineralizing the subsurface and surface of the affected area. In conclusion, the complex interaction between dental health, blood composition, and microbial variables emphasizes the significant influence of oral health on overall health. This study highlights the complex nature of this crucial link.

## Review

Thalassemia, sickle cell disease (SCD), iron deficiency anemia, and various other hematological disorders are a group of conditions that not only affect blood but can also have profound implications for oral health. These conditions, encompassing a spectrum of genetic and acquired disorders, are characterized by abnormalities in the blood's composition or function. While they primarily impact the hematological system, they can also lead to various oral health issues, including dental caries, periodontal disease, malocclusion, and other oral manifestations. Recognizing the dynamic relationship between these disorders and oral health is essential for delivering holistic care to the individuals affected. This article explores the relationships between hematological disorders and oral health, shedding light on how these conditions can influence oral health outcomes and emphasizing the importance of tailored dental management for individuals with such disorders.

Blood disorders

β-Thalassemia

**Figure 1:**
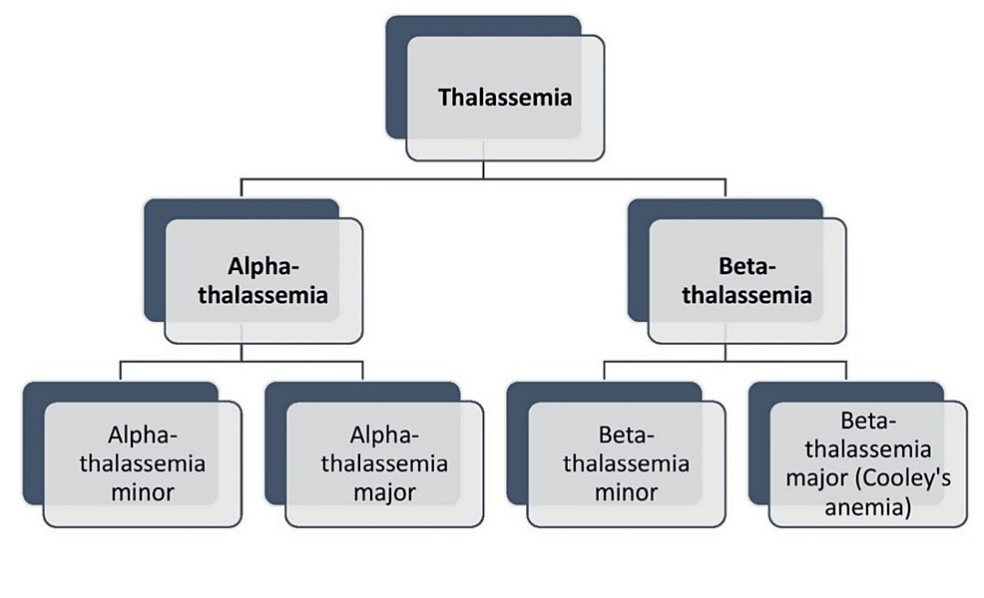
Classification of thalassemia Image credits: Gayatri Kale (author)

Thalassemia is a collection of inherited disorders that produce abnormal polypeptide chains in the hemoglobin (Hb) molecule. Thalassemia not only has detrimental effects on the individual's physical health but also has a psychosocial impact on the affected individual and their family. There are two primary types of thalassemia: α-thalassemia and β-thalassemia (Figure 1).

**Figure 2:**
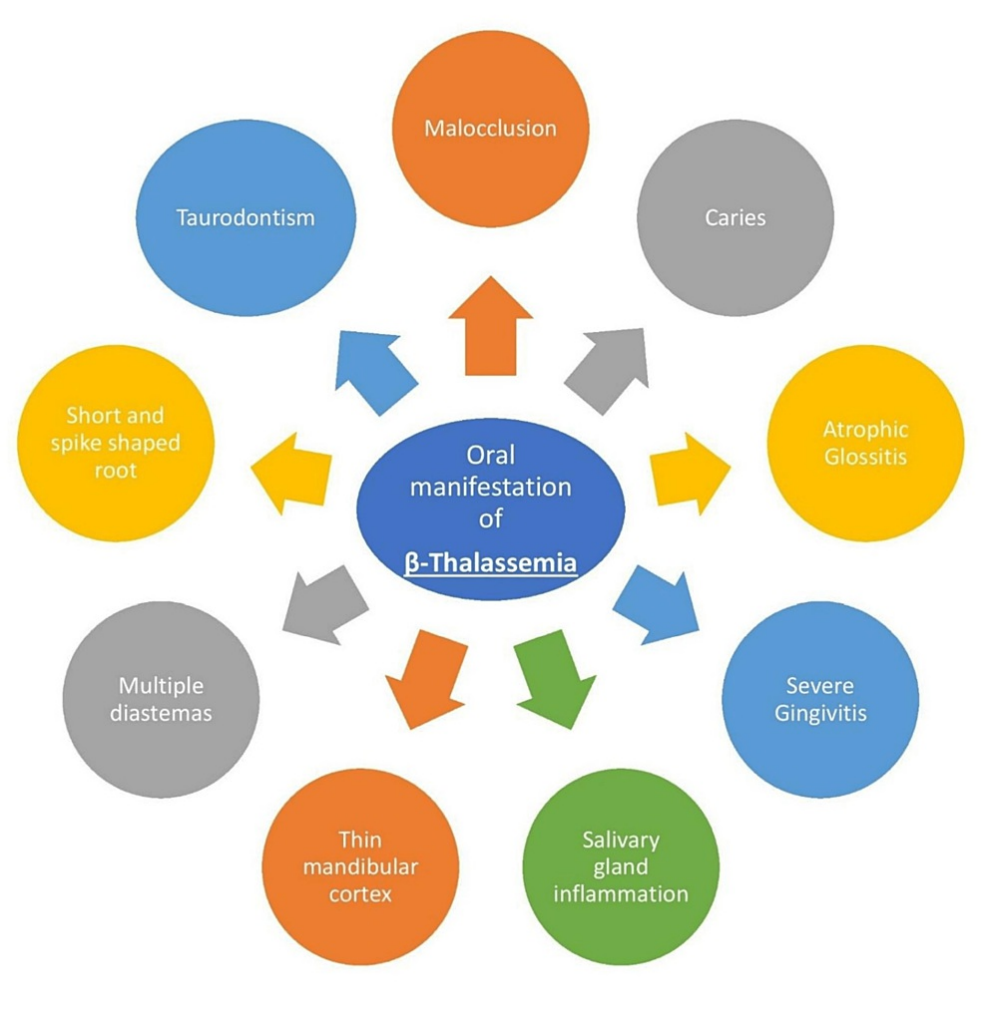
Oral and dental manifestations of β-thalassemia Image credits: Gayatri Kale (author)

Thalassemia is characterized based on both its clinical and genetic components. The prevalence of β-thalassemia major carriers is 2.78% [12]. The most severe clinical symptoms are displayed by β-thalassemia, often known as Cooley's anemia, whereas α-thalassemia appears milder and may not show any clinical indications [13]. Thalassemia major typically manifests clinically between 6 and 24 months after diagnosis. Infants with the condition do not thrive and become progressively paler. Thalassemia major is characterized by a clinical presentation that includes growth retardation, pallor, jaundice, decreased muscle tone, genu valgum, hepatosplenomegaly, leg ulcers, development of masses due to extramedullary hematopoiesis, and skeletal changes resulting from bone marrow enlargement. In infants under 2, severe microcytic anemia, moderate jaundice, and hepatosplenomegaly often raise suspicion of thalassemia major. Thalassemia intermedia, on the other hand, tends to manifest later with similar but less severe clinical symptoms. Other issues, such as feeding problems, constipation, agitation, recurrent fever attacks, and progressive abdominal enlargement due to spleen and liver enlargement, may also occur [14]. Thalassemia patients exhibit notably elevated occurrences of various oral manifestations, including sensations of burning in the oral mucosa, lingual varicosities, dry mouth, atrophic glossitis, and numbness in the oral mucosa. These symptoms are particularly prevalent among individuals with thalassemia [15]. Figure 2 enumerates the disease's oral and dental manifestations.

It was once believed that gingival irritation and dental caries were unrelated. In a study, Ventura asserted that the increase in caries prevalence is due to the endocrine disturbances brought on by thalassemia [16-17].

In a 2002 study, Al-Wahadni et al. investigated whether β-thalassemia major is related to worsening periodontal disease and dental caries. Plaque deposits, gingivitis, periodontitis, and dental caries were assessed in 61 affected patients and 63 healthy individuals aged 6-18 years using various periodontal indexes and the decayed, missing and filled teeth (DMFT) index, respectively. It was found that the thalassemia patient group's dental caries were higher than healthy patients [18]. In a 2014 study by Arora et al., the mean DMFT score was found to be higher among β-thalassemic patients [19]. In a study by Dhote et al. conducted in 2015, the DMFT index showed a slightly higher mean value in the thalassemia major group [20].

Sickle Cell Disease

SCD is an inherited hemoglobin disorder with a prevalence of approximately 10% [21]. It is caused due to abnormal hemoglobin known as hemoglobin S (HbS) that binds to red blood cells. This condition arises due to a specific point mutation in the β-globin chain of hemoglobin, where valine replaces glutamic acid in the β-globin chain. An epidemiological study on sickle cell disease was conducted at a rural hospital in central India by Kamble and Chaturvedi in 2000. The study included all patients admitted to the pediatric ward of Kasturba Hospital, Mahatma Gandhi Institute of Medical Sciences, Maharashtra, India, from August 1995 to July 1996. This study revealed an SCD prevalence of 5.7% (99 out of 1753 hospitalizations) [22].

Chronic hemolytic anemia, sickle cell crisis, susceptibility to bacterial infections, and slow tissue deterioration are the hallmarks of this condition [23]. SCD may present in various ways that might impact different body organs, including the kidney, lungs, musculoskeletal and cardiovascular systems, and so on [24]. Patients with sickle cell anemia frequently endure acute aplastic crises, intense pain that begins in infancy and lasts through childhood and with age, and symptomatic anemia [25]. Significantly decreased salivary flow, mental nerve neuropathy, elevated plaque levels, calculus deposition, radiographic abnormalities, enamel hypomineralization, delayed tooth eruption, dental caries, malocclusion, and hypercementosis are some of the common dental manifestations seen in people with SCD. Depapillation of the tongue, which leads to atrophic glossitis, is common in anemic patients. However, the prevalence of such symptoms among SCD patients remains uncertain. Unlike adults, children with SCD are more susceptible to developing periodontitis. Generally, individuals with SCD have an increased susceptibility to infections and periodontal diseases. The oral mucosa of SCD patients often exhibits pallor to yellowish discoloration due to the deposition of blood pigments due to hyperbilirubinemia resulting from erythrocytosis. Additionally, erythematous macular lesions, bleeding, and hemorrhage can affect the buccal and labial mucosa [26].

**Figure 3:**
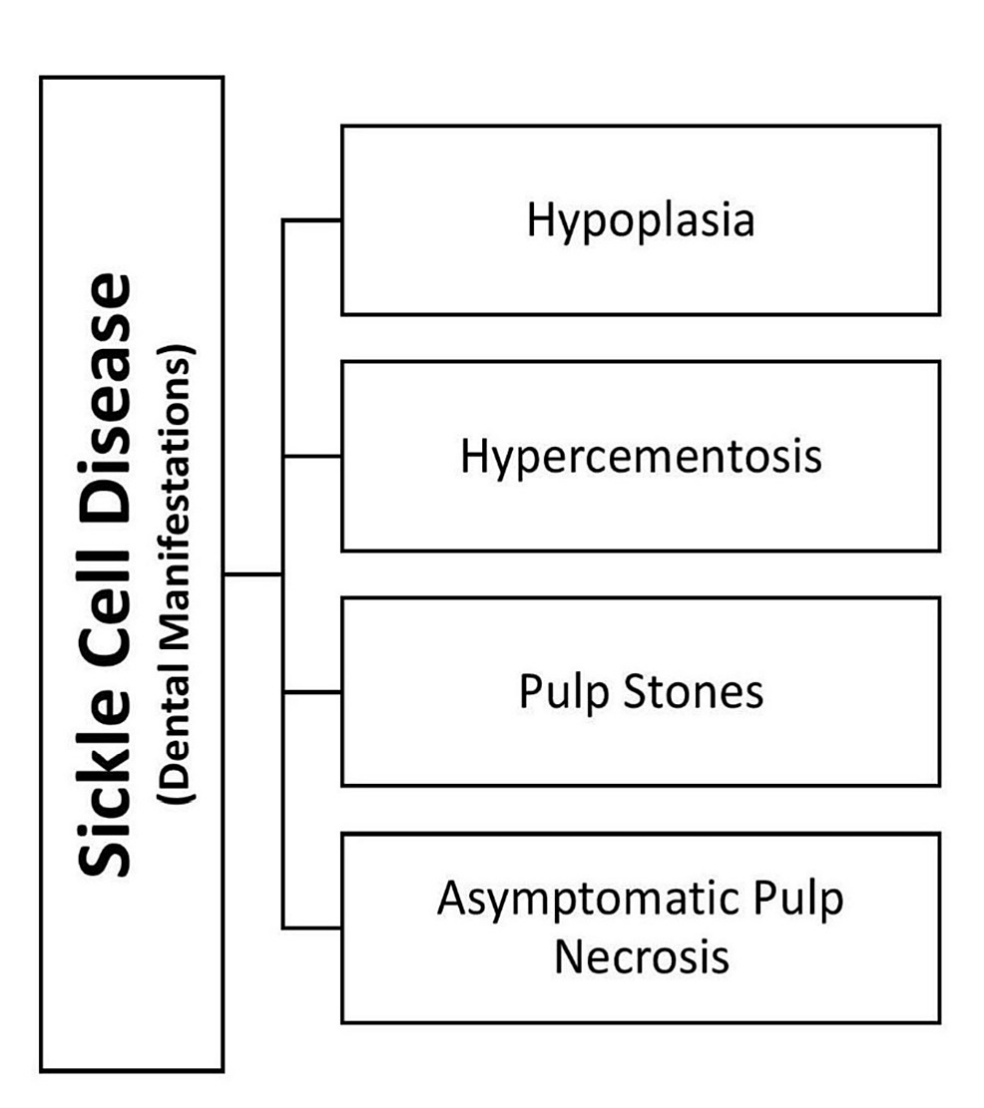
Dental manifestations of sickle cell disease Image credits: Gayatri Kale (author)

Fernandes et al., in 2015, found in a cross-sectional investigation that SCD also has degenerative effects on the oral cavity and dental tissues [27]. SCD has been linked to various dental abnormalities caused by blood vessel thrombosis (Figure 3).

On the other hand, SCD patients have a higher risk of dental complications due to changes in the dental structure, medications that contain sucrose, and increased susceptibility to infections, as found in an observational cross-sectional study by Medeiros et al. in 2018. Poor dental hygiene is always a risk factor for SCD [23].

In 2002, Laurence et al. conducted a pilot study on sickle cell anemia and dental caries. Low-income SCD patients had more decayed and fewer filled areas than patients without SCD. The study suggested that SCD patients with lower incomes may be more likely to experience dental caries and may have access to additional treatment options if caries are discovered. Despite the lack of statistically significant changes, these findings had clinical significance and encouraged further investigation through more in-depth future studies [28].

Iron Deficiency Anemia

The World Health Organization asserts that iron insufficiency is an anticipated dietary shortfall in developing and industrialized nations. The prevalence of iron deficiency anemia is more than 95% [29]. Iron is a crucial nutrient that aids in oxygen transportation. Iron deficiency anemia results from insufficient iron, which hinders hemoglobin synthesis. This deficiency may have short- and long-term impacts, including permanent dysfunctions of the growing central nervous system in children. In adults, a deficiency of iron that leads to anemia can have a wide range of detrimental health consequences. These include reduced work capacity, impaired regulation of body temperature, compromised immune function, gastrointestinal disturbances, and an increased susceptibility to *Helicobacter pylori *infection. Iron deficiency can lead to neurocognitive impairment, resulting in psychomotor and cognitive abnormalities in children. If left unaddressed, these issues can impact a child's learning ability. During pregnancy, iron deficiency anemia has long been linked to an elevated risk of low birth weight, preterm delivery, perinatal mortality, infant and young child mortality, and maternal mortality [30].

According to various research studies, early childhood caries is one of the oral symptoms of iron deficiency anemia, especially in young children under the age of five years. In children with iron deficiency, the impairment of salivary gland function leads to reduced buffering capacity due to decreased salivary secretions and lower salivary pH, which increases the risk of caries. Children under the age of five who experience caries and iron deficiency anemia have demonstrated early molar loss, potentially affecting dietary habits and resulting in various nutritional problems and malnutrition. Additionally, the reciprocal relationship holds in the case of iron deficiency anemia. Shaoul et al. noted in a study that iron deficiency in children leads to higher incidences of caries due to inadequate diets or diets with a higher consumption of cariogenic foods such as high-carbohydrate diets and beverages [31].

In conclusion, those with iron deficiency anemia should focus on their oral hygiene to reduce their chance of developing periodontal and dental caries. It is crucial to ensure children under 5 have a well-balanced diet that satisfies their nutritional needs and promotes healthy eating habits because this risk is particularly severe in young children. Such dietary modifications can be beneficial in reducing the likelihood of oral health issues in affected people.

Leukemia

Leukemia is a disorder primarily affecting white blood cells, characterized by an abnormal increase in immature or atypical leukocytes. Its prevalence ranges from 7.3% to 57.8% [32]. It is a malignant and progressive condition originating in tissues producing white blood cells, including bone marrow and other organs. This proliferation of aberrant blood cells is marked by compromised differentiation, regulation, and programmed cell death [33].

Solitary, dispersed, or clustered lesions on the chest, limbs, and head are among the possible systemic symptoms of leukemia; palms and soles are less frequently affected. The clinical symptoms of acute leukemia arise from the rapid development of bone marrow insufficiency. It leads to a severe clinical presentation characterized by high fever and gastrointestinal and pulmonary symptoms. Additionally, patients often experience progressively severe anorexia, muscle and joint pain, and episodes of hemorrhage. Leukemia can present orally as bleeding gums, oral mucosal ulcerations, petechiae, bone loss, and enlarged gingiva. Patients with leukemia with compromised immunity may develop secondary infections such as candidiasis and herpes simplex virus infections.

Increased tooth decay in the cervical region, tooth pain and mobility, and early tooth loss are additional typical dental symptoms of leukemia, especially in more severe instances. Cervical caries frequently result from the degenerating alveolar bone underneath and the periodontal ligament. Reduced oral hygiene, decreased salivation, which hinders oral cleaning, changes in oral microbiota, and dietary changes can all be blamed for the increased prevalence of dental caries [34].

Patients should be encouraged by effective treatment plans to practice good dental hygiene and use fluoride-containing dentifrices sparingly. Additionally, patients should be encouraged to maintain a diet low in carbs and dietary sucrose, eat non-sticky foods, and schedule routine dental examinations. Vigilance can significantly reduce dental caries while using sugar medications and maintaining oral hygiene. It is best to avoid giving children sugar-containing drugs right before bedtime [35].

Hemophilia

Hemophilia, often called the "royal disease," is a chromosomal bleeding disorder linked to the X chromosome. It is caused by a deficiency in either coagulation factors, precisely, factor VIII or factor IX. Hemophilia is categorized into two primary types based on the deficient factor: hemophilia A, resulting from a deficiency of factor VIII, and hemophilia B, resulting from a deficiency of factor IX. Both hemophilia A and B can be further classified into three categories based on the activity levels of these factors, which include mild, moderate, and severe forms. Hemophilia A is more common in males, with incidences accounting for approximately 80%-85% of cases, while hemophilia B is less frequent, occurring in roughly 10%-15% of cases.

Hemophilia's clinical manifestation can result in a range of issues, including pregnancy-associated and neonatal bleeding, heightened susceptibility to bruising, excessive bleeding following minor injuries or surgeries, spontaneous bleeding in diverse body regions, inflammation of the joints, muscle hematomas, and the formation of pseudotumors. Hemophilia patients are prone to musculoskeletal complications such as chronic hemophilic arthropathy, synovitis, contractures, developing inhibitors against factor VIII, and pseudotumor formation. Hemophilia is marked by bleeding from various sites, often observed in the mouth as gingival bleeding and post-extraction hemorrhages. The frequency of oral bleeding incidents varies depending on the severity of the individual's hemophilia, potentially leading to multiple occurrences throughout their life. [36]. A greater incidence of dental caries and other oral problems is present in people with inherited coagulation abnormalities, such as hemophilia, since they are less likely to adopt regular oral hygiene routines to stop bleeding. Another issue that could occur during dental treatment is the administration of a local anesthetic since factor replacement is required when executing inferior alveolar blocks because of the rich vasculature and the potential for hematoma development in sites like the retromolar or pterygoid regions [37].

Numerous investigations of the oral health of hemophiliacs have found a high frequency of dental problems and poor oral hygiene. Dental operations are frequently avoided because people fear having long-lasting bleeding, which increases the likelihood that a treatment won't work. Those who have inherited blood issues must put their oral health first. The oral health requirements of these individuals can be successfully guided and managed by a combination of several dental visits and routine consultations with a hematologist, physician, and dentist [38].

Plummer-Vinson Syndrome

Plummer-Vinson syndrome (PVS) is documented to be more frequent in females than males, with a notable ratio of approximately 8:1 [39]. PVS is defined by post-cricoid dysphagia, upper esophageal webs, and iron deficiency, with at least five out of nine iron insufficiency criteria being met. In the United Kingdom, a similar clinical condition with identical symptoms is called the "Paterson-Kelly syndrome" [40]. PVS primarily affects women in their 30s-60s, with a lower incidence observed in males [41-42]. Common symptoms of anemia reported by patients include weakness, pallor, fatigue, and tachycardia. Additional features that may be observed include esophagitis, cardiospasm, achlorhydria, nail abnormalities like koilonychia or clubbing, enlargement of the spleen and thyroid, spleen tumors, dermatitis, seborrhea, hyperkeratosis, conjunctivitis, keratitis, blepharitis, and visual disturbances [43].

People with PVS are more likely to develop fibrosis, and extending these fibrotic areas can cause pain when speaking, eating, or maintaining good dental hygiene. Consequently, they can develop periodontal disease, increased plaque accumulation, cavities, and poor oral hygiene [44-45].

Erythroblastosis Fetalis

Erythroblastosis fetalis refers to the hemolytic anemia that neonates experience due to blood incompatibility between the mother and fetus [46]. It occurs in about 15% of pregnancies [47]. Alongside symptoms like ascites, hepatosplenomegaly, edema, and jaundice soon after birth, severely affected newborns appear pale and frail due to elevated bilirubin levels [48]. Dental findings include varying degrees of enamel abnormalities during development. Examples of enamel defects include hypoplasia confined to primary dentition or issues with the cusps of permanent first molars. The timing of metabolic disruption and the severity of dental problems are interconnected.

Cullen conducted a case study on a three-and-a-half-year-old White male with a prenatal diagnosis of erythroblastosis fetalis due to Kell incompatibility. No caries were noted in the patient. Enamel hypoplasia was observed on maxillary and mandibular incisors, canines, first molars, and second molars. Nursing caries syndrome was diagnosed as the child in this study was breastfed until the age of 2 and exhibited chipped and yellow enamel. Postnatally generated enamel was found to possess standard color, texture, and quantity upon careful examination. No clinical caries were caused due to the hypoplasia or thalassemia in the child. However, the child's parents were cautioned about the potential higher risk of cavities due to enamel hypoplasia [49]. Nevertheless, there is no direct evidence linking erythroblastosis fetalis to dental caries.

Fanconi Anaemia

Fanconi anemia (FA) is a genetically and phenotypically diverse recessive disorder characterized by congenital abnormalities, progressive pancytopenia, and susceptibility to solid tumors and hematologic malignancies. FA exists in individuals of all racial and ethnic backgrounds, with a reported carrier frequency of 1 in 300. The clinical presentation of FA includes symptoms such as pancytopenia, hyperpigmentation, skeletal malformations, reduced stature, urogenital abnormalities, and a tendency for familial occurrence [50]. The most common oral symptoms in those with this condition are gingivitis and periodontitis. Poor oral hygiene and biofilm formation contribute to the progression of these conditions in affected individuals. Factors such as regular sugar consumption and specific cariogenic bacteria may lead to the development of dental caries. Low economic status and limited access to oral health care could also play a role [51].

In a 2007 study, Tekcicek et al. investigated the oral health of children between the ages of 2 and 18 with Fanconi anemia. The study found that 35% of the participants had dental caries [52]. A case study conducted in 2016 by Goswami et al. focused on a five-year-old male child with FA. The intraoral examination revealed several carious lesions. The patient underwent multiple symptomatic restorations during dental visits due to poor oral hygiene maintenance, resulting in frequent oral infections and ulcers [53].

Cyclic Neutropenia

Cyclic neutropenia is a benign hematologic condition characterized by periodic episodes of severe neutropenia that occur in cycles of 21 days. Epidemiological studies have revealed that the prevalence of neutropenia (having a neutrophil count of less than 1.5 g/L) is approximately 4.5% among individuals of Black ethnicity and 0.8% among those of White ethnicity [54]. Additionally, some blood cells exhibit cyclical variations. The exact cause of cyclic neutropenia remains unidentified. The condition manifests suddenly, with symptoms typically appearing in infancy or early childhood. However, it can also develop in adult patients and be accompanied by clonal proliferation of large granular cells. Symptoms typically emerge during infancy or childhood in the majority of cases. The diagnosis often arises when patients exhibit neutropenia episodes alongside a history of recurrent upper respiratory tract infections. In some instances, patients may develop mucosal ulcers in the intestinal tract, specifically in the colon, rectum, and anus, potentially leading to bacteremia from these sites [55]. Individuals with severe neutropenia, defined as a neutrophil count below 500/mm³, experience symptoms such as pharyngitis, stomatitis, oral ulcers, and enlarged lymph nodes. Serious infections can also occur. Severe gingivitis and oral ulceration during neutropenia are oral symptoms of cyclic neutropenia.

In a 1996 study by Pernu et al., salivary variables and the incidence of dental caries were investigated. While no conclusive evidence linking tooth caries to the condition was found, it was stated that poor oral hygiene could contribute to caries [56].

Acute Lymphoblastic Leukemia

Acute lymphoblastic leukemia (ALL) accounts for 12% of all leukemia cases and is estimated to have a global incidence ranging from 1 to 4.75 cases per 100,000 people [57]. ALL is the malignant transformation and proliferation of lymphoid progenitor cells in the bone marrow, blood, and extramedullary locations. While 80% of reported cases of ALL are seen in young individuals, the disease is equally dreadful when it affects adults. Malignant, poorly differentiated lymphoid cells accumulate in the bone marrow, peripheral blood, and extramedullary sites, reflected in most ALL clinical manifestations. Constitutional symptoms and bone marrow failure symptoms can result in anemia, thrombocytopenia, and leukopenia, sometimes presenting as nonspecific. Common symptoms include fever, weight loss, night sweats, bleeding, fatigue, dyspnea, and infection susceptibility. Extramedullary involvement is frequent and can lead to patients' lymphadenopathy, splenomegaly, or hepatomegaly [58].

In the oral cavity, the most typical symptoms of leukemia include gingival bleeding, hyperplasia, opportunistic infections, and bone changes [59]. A study conducted by Pajari et al. revealed that children who received central nervous system radiation had higher DMFT scores (7.13) compared to children who only underwent chemotherapy (3.4), and controls (1.8). Studies on the lifetime incidence of caries indicated that children with ALL developed 2.7 new caries lesions per year during treatment, in contrast to new lesions in controls [60].

Guidelines for maintaining oral hygiene in hematological disorders

Patients with hematological disorders face an increased risk of dental problems, but there are crucial guidelines they should adhere to to maintain optimal oral health. First, maintaining a rigorous oral hygiene routine is essential, involving brushing teeth at least twice daily with a fluoride toothpaste, utilizing a soft-bristled toothbrush, and replacing it every three to four months [61]. Additionally, it is advisable to moderate sugar intake, as excessive sugar consumption can contribute to dental caries. Sugary medications should ideally be avoided before bedtime to limit prolonged sugar exposure. Regular dental check-ups and cleanings are crucial for monitoring oral health and applying preventive treatments like fluoride applications and dental sealants [62]. For those experiencing dry mouth due to their condition, staying hydrated and considering sugar-free gum or lozenges may help, and a dentist could recommend artificial saliva products. When mobility is limited due to the hematological disorder, discussions with the dentist about the frequency of scaling and root planing become necessary to prevent plaque and calculus buildup. In early childhood caries or rampant caries, fluoride applications can strengthen tooth enamel, while minimally invasive dental treatments like laser-assisted procedures are encouraged for caries detection and treatment. Maintaining open communication with healthcare providers, including hematologists, general physicians, and dentists, through regular consultations is vital to effectively manage the hematological condition and oral health. By diligently following these guidelines, individuals with hematological disorders can take proactive measures to safeguard their oral health, diminish the risk of dental complications, and enhance their overall quality of life.

## Conclusions

Hematological diseases significantly impact oral hygiene, leading to compromised oral health due to physical and mental health factors. This results in heightened plaque and calculus accumulation, increasing the susceptibility to dental caries. Reduced salivary flow, often observed in hematological disorders, further exacerbates the situation. While saliva's natural cleansing and pH regulation roles are essential for oral health, its altered quantity in individuals with these conditions leads to a higher prevalence of dental caries than their healthy counterparts. The incidence of caries is closely tied to patient therapy and oral hygiene practices, with severe cases potentially requiring tooth extraction and affecting dietary habits and overall well-being.

## References

[1] Selwitz RH, Ismail AI, Pitts NB (2007). Dental caries. Lancet.

[2] Peres MA, Macpherson LM, Weyant RJ (2019). Oral diseases: a global public health challenge. Lancet.

[3] Pitts NB, Zero DT, Marsh PD (2017). Dental caries. Nat Rev Dis Primers.

[4] Wade WG (2013). The oral microbiome in health and disease. Pharmacol Res.

[5] Christian B, Ummer-Christian R, Blinkhorn A, Hegde V, Nandakumar K, Marino R, Chattopadhyay A (2019). An epidemiological study of dental caries and associated factors among children residing in orphanages in Kerala, India: Health in Orphanages Project (HOPe). Int Dent J.

[6] Gupta B, Bray F, Kumar N, Johnson NW (2017). Associations between oral hygiene habits, diet, tobacco and alcohol and risk of oral cancer: a case-control study from India. Cancer Epidemiol.

[7] Janakiram C, Mehta A, Venkitachalam R (2020). Prevalence of periodontal disease among adults in India: a systematic review and meta-analysis. J Oral Biol Craniofac Res.

[8] Watanabe Y, Okada K, Kondo M, Matsushita T, Nakazawa S, Yamazaki Y (2020). Oral health for achieving longevity. Geriatr Gerontol Int.

[9] Sivapathasundharam B, Raghu AR (2009). Dental caries. Shafer's Textbook of Oral Pathology, 6th Edition.

[10] Teughels W, Laleman I, Quirynen M, Jakubovics N, Ambalvanan N (2018). Biofilm and periodontal microbiology. Newman and Carranza's Clinical Periodontology. Third South Asia Edition.

[11] Sapkota J, Sharma M, Jha B, Bhatt CP (2019). Prevalence of Staphylococcus aureus isolated from clinical samples in a tertiary care hospital: a descriptive cross-sectional study. JNMA J Nepal Med Assoc.

[12] Mohanty D, Colah RB, Gorakshakar AC (2013). Prevalence of β-thalassemia and other haemoglobinopathies in six cities in India: a multicentre study. J Community Genet.

[13] Al-Wahadni AM, Taani DQ, Al-Omari MO (2002). Dental diseases in subjects with beta-thalassemia major. Community Dent Oral Epidemiol.

[14] Galanello R, Origa R (2010). Beta-thalassemia. Orphanet J Rare Dis.

[15] Wang YP, Chang JY, Wu YC, Cheng SJ, Chen HM, Sun A (2013). Oral manifestations and blood profile in patients with thalassemia trait. J Formos Med Assoc.

[16] Helmi N, Bashir M, Shireen A, Ahmed IM (2017). Thalassemia review: features, dental considerations and management. Electron Physician.

[17] Pradhan M, Sudha P (2020). Evaluation of clinicoradiological orofacial structures in children with β-thalassemia major. J South Asian Assoc Pediatr Dent.

[18] Nabi AT, Muttu J, Chhaparwal A, Mukhopadhyay A, Pattnaik SJ, Choudhary P (2022). Implications of β-thalassemia on oral health status in patients: a cross-sectional study. J Family Med Prim Care.

[19] Arora R, Malik S, Arora V, Malik R (2014). Comparison of dental caries prevalence in Β-thalassemia major patients with their normal counterparts in Udaipur. Am Int J Res Form Appl Nat Sci.

[20] Dhote V, Thosar N, Baliga S (2015). Evaluation of oral hygiene status and salivary biochemistry of patients with thalassemia major: a clinical study. IOSR J Dent Med Sci.

[21] Wankhade V, Andhale RB, Lodha S (2013). Diverse clinical manifestations in sickle cell anemia: study in district Amravati, MS India. J Blood Disorders Transf.

[22] Kamble Á, Chaturvedi P (2000). Epidemiology of sickle cell disease in a rural hospital of central India. Indian Pediatr.

[23] Medeiros ML, Mendes LL, Lopes SL (2018). Analysis of oral health conditions and risk factors for dental caries in patients with sickle cell disease. Rev Gaúcha Odontol.

[24] Yue H, Xu X, Liu Q, Li X, Jiang W, Hu B (2020). Association between sickle cell disease and dental caries: a systematic review and meta-analysis. Hematology.

[25] Konotey-Ahulu FD (1974). The sickle cell diseases: clinical manifestations including the sickle crisis. Arch Intern Med.

[26] Al-Jafar H, Dashti H, Al-Haddad SJ, Al-Qattan S, Al-Ramzi A (2016). Dental alterations in sickle cell disease. J Dent Oral Care Med.

[27] Fernandes ML, Kawachi I, Corrêa-Faria P, Pattusi MP, Paiva SM, Pordeus IA (2015). Caries prevalence and impact on oral health-related quality of life in children with sickle cell disease: cross-sectional study. BMC Oral Health.

[28] Laurence B, George D, Woods D (2006). The association between sickle cell disease and dental caries in African Americans. Spec Care Dentist.

[29] Chellan R, Paul L (2010). Prevalence of iron-deficiency anaemia in India: results from a large nationwide survey. J Popul Soc Stud.

[30] Clark SF (2008). Iron deficiency anemia. Nutr Clin Pract.

[31] Shaoul R, Gaitini L, Kharouba J, Darawshi G, Maor I, Somri M (2012). The association of childhood iron deficiency anaemia with severe dental caries. Acta Paediatr.

[32] Radhakrishnan VS, Agrawal N, Bagal B, Patel I (2021). Systematic review of the burden and treatment patterns of adult and adolescent acute lymphoblastic leukemia in India: comprehending the challenges in an emerging economy. Clin Lymphoma Myeloma Leuk.

[33] Prasad SR, Anitha Anitha, Jayaram R, Pai A, Yaji AY, Jambunath U, Yadav K (2018). Oral manifestations of myeloid neoplasms and acute leukemia - a diagnostic perspective. Hematol Transfus Int J.

[34] Mathur VP, Dhillon JK, Kalra G (2012). Oral health in children with leukemia. Indian J Palliat Care.

[35] Wagner G, Fenchel K, Back W, Schulz A, Sachse MM (2012). Leukemia cutis - epidemiology, clinical presentation, and differential diagnoses. J Dtsch Dermatol Ges.

[36] Shastry SP, Kaul R, Baroudi K, Umar D (2014). Hemophilia A: dental considerations and management. J Int Soc Prev Community Dent.

[37] Zaliuniene R, Peciuliene V, Brukiene V, Aleksejuniene J (2014). Hemophilia and oral health. Stomatologija.

[38] Norbutaev A, Shamsiev M, Nazarova N (2021). Clinical and functional changes in hard tissues of teeth in patients with hemophilia. Am J Med Sci Pharm Res.

[39] Mangalagiri GD (2019). Clinical perspective on Plummer-Vinson-syndrome in south India. Nat J Med Dent Res.

[40] Atmatzidis K, Papaziogas B, Pavlidis T, Mirelis Ch, Papaziogas T (2003). Plummer-Vinson syndrome. Dis Esophagus.

[41] Bakshi SS (2015). Plummer Vinson syndrome - is it common in males?. Arq Gastroenterol.

[42] Karthikeyan P, Aswath N, Kumaresan R (2017). Plummer Vinson syndrome: a rare syndrome in male with review of the literature. Case Rep Dent.

[43] Hasan S, Khan NI, Siddiqui A (2013). Plummer Vinson syndrome - a premalignant condition - an overview of literature. J Dent Med Sci.

[44] Priya NK, Shruthy R, Ramakrishna A, Sowmya NK, Madhushankari GS (2016). Enigma of oral potentially malignant disorders - a brief overview. J Adv Clin Res Insights.

[45] Hasan S, Saeed S (2015). Xeroderma pigmentosum-a rare genodermatosis: overview of literature. J Pigment Disord.

[46] Kline BS (1949). The pathogenesis of erythroblastosis fetalis. Blood.

[47] Nassar GN, Wehbe C (2023). Erythroblastosis fetalis. StatPearls [Internet].

[48] Wiener AS (1946). Pathogenesis of erythroblastosis fetalis. Proc Soc Exp Biol Med.

[49] Cullen CL (1990). Erythroblastosis fetalis produced by Kell immunization: dental findings. Pediatr Dent.

[50] Auerbach AD (2009). Fanconi anemia and its diagnosis. Mutat Res.

[51] D'Agulham AC, Chaiben CL, Lima AA, Torres-Pereira CC, Machado MA (2014). Fanconi anemia: main oral manifestation. Rev Gaúch Odontol.

[52] Tekcicek M, Tavil B, Cakar A, Pinar A, Unal S, Gumruk F (2007). Oral and dental findings in children with Fanconi anemia. Pediatr Dent.

[53] Goswami M, Bhushan U, Goswami M (2016). Dental perspective of rare disease of Fanconi anemia: case report with review. Clin Med Insights Case Rep.

[54] Donadieu J, Beaupain B, Mahlaoui N, Bellanné-Chantelot C (2013). Epidemiology of congenital neutropenia. Hematol Oncol Clin North Am.

[55] Dale DC, Hammond WP IV (1988). Cyclic neutropenia: a clinical review. Blood Rev.

[56] Pernu HE, Pajari UH, Lanning M (1996). The importance of regular dental treatment in patients with cyclic neutropenia. Follow-up of 2 cases. J Periodontol.

[57] Redaelli A, Laskin BL, Stephens JM, Botteman MF, Pashos CL (2005). A systematic literature review of the clinical and epidemiological burden of acute lymphoblastic leukaemia (ALL). Eur J Cancer Care (Engl).

[58] Terwilliger T, Abdul-Hay M (2017). Acute lymphoblastic leukemia: a comprehensive review and 2017 update. Blood Cancer J.

[59] Morais EF, Lira JA, Macedo RA, Santos KS, Elias CT, Morais M (2014). Oral manifestations resulting from chemotherapy in children with acute lymphoblastic leukemia. Braz J Otorhinolaryngol.

[60] Pajari U, Ollila P, Lanning M (1995). Incidence of dental caries in children with acute lymphoblastic leukemia is related to the therapy used. ASDC J Dent Child.

[61] Choo A, Delac DM, Messer LB (2001). Oral hygiene measures and promotion: review and considerations. Aust Dent J.

[62] Levy SM, Kiritsy MC, Warren JJ (1995). Sources of fluoride intake in children. J Public Health Dent.

